# Génotypage par pcr-rflp de *pfcrt* et *pfmdr1* sur des isolats de *plasmodium falciparum* collectés chez des enfants de Vatomandry, Madagascar

**DOI:** 10.48327/mtsi.v2i2.2022.198

**Published:** 2022-06-16

**Authors:** Élisabeth RAVAOARISOA, Voahangy Hanitriniaina Isabelle ANDRIANARANJAKA, Aina David RAMANANTSAHALA, Tovonahary Angelo RAKOTOMANGA, Fanomezantsoa RALINORO, Rianasoambolanoro RAKOTOSAONA, Ranjàna Hanitra RANDRIANARIVO, Danielle Aurore Doll RAKOTO, Victor JEANNODA, Arsène RATSIMBASOA

**Affiliations:** 1 Université d'Antananarivo Faculté des sciences, Mention Biochimie fondamentale et appliquée, Madagascar; 2 Programme national de lutte contre le paludisme, Ministère de la Santé publique, Antananarivo, Madagascar; 3 Centre national d'application de recherches pharmaceutiques, Ambodivoanjo, Antananarivo, Madagascar; 4 Faculté de médecine, Université de Fianarantsoa, Madagascar

**Keywords:** Antipaludique, Enfants, *Plasmodium falciparum*, *Pfmdr1*, *Pfcrt*, Résistance, Vatomandry, Madagascar, Océan Indien, Antimalarial, Children, *Plasmodium falciparum*, *Pfmdr1*, *Pfcrt*, Resistance, Vatomandry, Madagascar, Indian Ocean

## Abstract

**Contexte:**

La surveillance de l'efficacité thérapeutique et des marqueurs génétiques de la résistance aux antipaludiques est primordiale afin de détecter le plus tôt possible l’émergence des parasites potentiellement résistants. Dans ce contexte, notre étude a pour objectif de réaliser le typage du gène *Plasmodium falciparum chloroquine resistance transporter* ou *Pfcrt* et *Plasmodium falciparum multidrug resistant gene 1* ou *Pfmdr1* sur des isolats provenant des enfants du district de Vatomandry.

**Méthodologies:**

Au total, 142 isolats de *P. falciparum* collectés lors d'un dépistage actif du paludisme chez des enfants âgés de moins de 15 ans, entre février et mars des années 2016 et 2017 à Vatomandry, ont été analysés. Le typage du codon K76T du gène *Pfcrt* et du codon N86Y du gène *Pfmdr1* a été ensuite effectué par la technique de polymérisation en chaîne suivie de digestion enzymatique *(restriction fragment length polymorphism)* ou PCR-RFLP.

**Résultats:**

Le taux de succès d'amplification des gènes *Pfcrt* et *Pfmdr1* était faible, de l'ordre de 27 % et 39 % respectivement. La prévalence des isolats mutants pour le codon K76T de *Pfcrt* était de 2,6 % [intervalle de confiance à 95 % (IC95%) : 0,1 - 15,0 %] et de 36 % [IC95% : 23,7 - 49,7 %] pour le codon N86Y du gène *Pfmdr1.*

**Conclusion:**

Notre étude a mis en évidence la présence d'isolats portant à la fois la mutation au niveau du codon K76T de *Pfcrt* et N86Y de *Pfmdr1.* Bien que le taux de mutation que nous avons observé soit faible, d'autres études méritent d’être effectuées afin de suivre l’évolution de ces marqueurs dans le temps et dans l'espace à Madagascar.

## Abstract (en)

**Background:**

Malaria is a parasitic disease caused by a hematozoan of the genus *Plasmodium.* Early diagnosis followed by effective treatment is one of the keys to control this disease. In Madagascar, after more than 60 years of use for the treatment of uncomplicated malaria, chloroquine (CQ) was abandoned in favor of artesunate + amodiaquine (ASAQ) combination because of high prevalence of CQ treatment failure. Surveillance based on the assessment of therapeutic efficacy and genetic markers of resistance to antimalarials is therefore essential in order to detect the emergence of potentially resistant parasites as early as possible. In this context, our study aimed to genotype the *Plasmodium falciparum* chloroquine resistance transporter gene or *Pfcrt* and *Plasmodium falciparum* multidrug resistance gene 1 or *Pfmdr1* in isolates collected from children in the district of Vatomandry.

**Methods:**

A total of 142 *P. falciparum* isolates collected during active case detection of malaria in children under 15 years old, between February and March of 2016 and 2017 in Vatomandry district, were analyzed. *Pfcrt* (K76T codon) and *Pfmdr1* (N86Y codon) genotyping was carried out by polymerase chain reaction followed by enzymatic digestion (restriction fragment length polymorphism) or PCR-RFLP.

**Results:**

The successful rates of amplification of *Pfcrt* and *Pfmdr1* genes were low, around 27% and 39% respectively. The prevalence of isolates carrying the mutant *Pfcrt* K76T codon and the mutant *Pfmdr1* N86Y codon was 2.6% [95% confidence interval (95% CI): 0.1 - 15.0%] and 36% [95% CI: 23.7 - 49.7%] respectively.

**Conclusion:**

Despite the limited number of samples analyzed, our study highlighted the circulation of isolates carrying both the mutant *Pfcrt* K76T and *Pfmdr1* N86Y alleles. Although the prevalence of mutations in *Pfcrt* and *Pfmdr1* genes that we observed was low, other studies should be carried out in order to follow the evolution of these markers in time and space. The use of more sensitive methods will better characterize *P. falciparum* strains circulating in Madagascar. Artesunate-amodiaquine is used as a first-line treatment for uncomplicated malaria in the country; it is also crucial to monitor the other codons, i.e. 184 and 1246 of the *Pfmdr1* gene, implicated in the resistance of *P. falciparum* to amodiaquine in Africa.

## Introduction

Le paludisme demeure un problème majeur de santé publique dans le monde. En 2020, 241 millions de cas ont été enregistrés dont 95 % dans la seule région africaine. Le nombre de décès a atteint 627 000 dont 77 % chez les enfants de moins de 5 ans. Le paludisme causé par *Plasmodium falciparum* est le responsable de la forme grave voire mortelle [22]. Un diagnostic précoce et fiable [8, 29] suivi d'un traitement efficace est l'une des clés pour lutter contre cette maladie. Cependant, la lutte contre le paludisme se heurte actuellement à la diffusion de souches résistantes aux antipaludiques utilisés. À Madagascar, la chloroquine (CQ), considérée comme peu toxique et d'un coût abordable, était utilisée comme traitement des accès palustres non compliqués jusqu'en 2005 [3, 27]. Toutefois, des études ont montré plusieurs cas d’échecs thérapeutiques vis-à-vis de la CQ [4, 17], ce qui justifie le retrait de cette molécule au profit de la combinaison d'artésunate-amodiaquine (ASAQ) pour le traitement en première intention et l'artéméther-luméfantrine (AL) en seconde intention [24]. La présence de mutations ou la surexpression du gène impliqué dans la résistance confère une baisse de la sensibilité ou la résistance des parasites aux antipaludiques [15, 25]. Ainsi, plusieurs marqueurs moléculaires impliqués dans la résistance aux antipaludiques ont été décrits [25]. Pour les médicaments utilisés en combinaison avec les dérivés d'artémisinine dans le schéma thérapeutique à Madagascar, les mutations au niveau des codons 86Y, Y184 et 1246Y de *Pfmdr1* et 76T de *Pfcrt* sont associées à la diminution de la sensibilité de l'AQ tandis que N86, 184F et D1246 de *Pfmdr1* et K76 de *Pfcrt* sont plutôt liées à la résistance à la luméfantrine [23]. Bien que la chloroquine ne soit plus utilisée à Madagascar, les mutations spécifiques au niveau du gène *Pfcrt* (K76T) et *Pfmdr1* (N86Y) sont utilisées comme des marqueurs moléculaires pour la surveillance de la chloroquino-résistance et dans la modulation des réponses vis-à-vis des autres médicaments partenaires associés à l'artésunate [7, 23, 30]. La surveillance de l'efficacité thérapeutique, y compris les marqueurs génétiques de la résistance aux antipaludiques, est alors primordiale afin de détecter le plus tôt possible l’émergence ou la diffusion des parasites potentiellement résistants afin d'orienter les politiques thérapeutiques. Dans ce contexte, notre étude a pour objectif de réaliser le typage des gènes *Pfcrt* et *Pfmdr1* de *P. falciparum* sur des isolats provenant des enfants du district de Vatomandry.

## Méthodes

### Site, population d’étude et échantillons analysés

Les échantillons analysés ici ont été déjà caractérisés comme mono-infection à *P. falciparum* lors d'une précédente étude réalisée au sein du laboratoire de parasitologie du Programme national de lutte contre le paludisme (PNLP) de Madagascar [26]. Ils ont été collectés lors du dépistage actif (détection des cas de paludisme par des agents de santé au niveau des communautés, [21]) chez des enfants de moins de 15 ans à Vatomandry entre février et mars des années 2016 et 2017, période correspondant au pic de la transmission. Les enfants dont les tuteurs ont été consentants et qui n'avaient pas pris d'antipaludiques

1 mois avant l'enquête ont été inclus dans cette étude. Pour chaque individu, le test de diagnostic rapide ou TDR basé sur la détection de la protéine riche en histidine
2 de *P. falciparum* ou PfHRP2 et du lactate déshydrogénase du genre *Plasmodium* ou pan-LDH (SD BIOLINE Malaria Ag Pf/

Pan, Standard Diagnostics) a été réalisé sur le terrain pour le diagnostic biologique du paludisme. Des gouttes de sang capillaire ont été collectées sur papier buvard pour la détection et l'identification de l'espèce plasmodiale par la PCR nichée et pour le génotypage de *Pfcrt* et *Pfmdr1.* Ces isolats ont été conservés à -20 °C jusqu’à leur utilisation.

**Figure 1:**
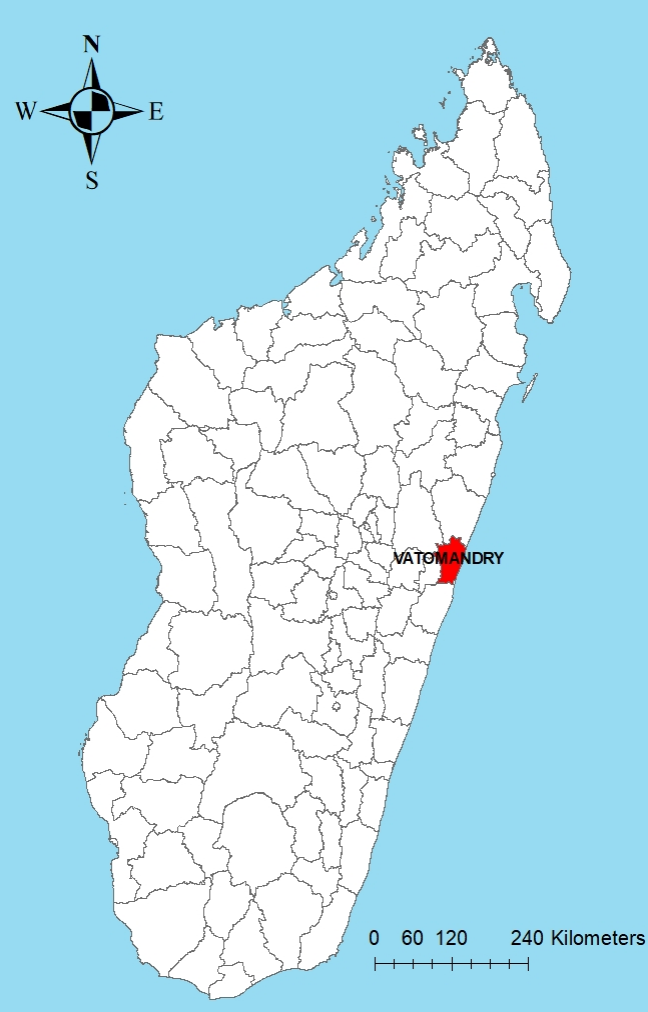
Site d’étude (Programme national de lutte contre le paludisme, Madagascar) Study site (National Malaria Control Program, Madagascar)

Vatomandry est situé sur le littoral Est de Madagascar, province de Toamasina, dans la région Atsinanana, à 265 km à l'est de la capitale Antananarivo (Fig. 1). La transmission des plasmodies y est forte et pérenne [10, 18].

### Diagnostic de *P. falciparum* par la PCR nichée

L'extraction de l'ADN par InstaGeneTM Matrix (BioRad™ Laboratories Inc., Marnesla-Coquette, France) a été effectuée selon le protocole recommandé par le fabricant. Les échantillons de sang sur papier buvard ont été découpés, récupérés dans des tubes stériles de 1,5 ml et incubés avec

1 ml de saponine 0,5 % pendant une nuit à température ambiante. La préparation a ensuite été centrifugée à 7 000 g pendant 3 min. Le culot obtenu a été re-suspendu avec 500 µl de PBS 1× puis centrifugé à 7 000 g pendant 3 min. Deux cents microlitres (200 µl) d'InstaGeneTM Matrix ont été ajoutés au culot. Le mélange a été incubé pendant 30 min à 56 °C puis à 100 °C pendant 8 min. Après une centrifugation à 21 000 g pendant 3 min, 180 µl de surnageant contenant l'extrait d'ADN ont été transférés dans un nouveau tube, puis conservés à -20 °C jusqu’à son utilisation.

La PCR nichée décrite par Snounou et ses collaborateurs [33] basée sur la détection du gène de l'ARN ribosomal 18S de *Plasmodium* spp. a été utilisée. La première étape a consisté à amplifier une région de 1200 paires de bases (pb). Deux microlitres (2 du produit d'amplification de la première PCR ont été réamplifiés avec l'amorce spécifique de *P. falciparum.* Les produits d'amplification de la seconde PCR ont été visualisés sous un rayon ultraviolet (UV) (GelDoc^®^; BioRad Laboratories Inc., Marnes-la-Coquette, France) après l’électrophorèse sur gel d'agarose contenant

2 % de bromure d’éthidium (BET). La taille des fragments a été estimée en la comparant avec le marqueur de poids moléculaire

100 pb (lot 100pb BPB 0230). La présence d'une bande de 206 pb confirmait l'infection à *P. falciparum.* Les échantillons confirmés positifs à *P. falciparum* ont été analysés pour le génotypage de *Pfcrt* et *Pfmdr1.*

### Génotypage du codon 76 de *Pfcrt*

Le fragment du gène *Pfcrt* contenant le codon 76 a été amplifié par la technique de la PCR nichée. La première PCR amplifie une région de 369 pb avec le couple d'amorces externes CRTP1 (5’-CCGTTAATAATAAA-TACACGCAG-3’) et CRTP2 (5’-CGGA-TGTTACAAAACTATAGTTACC-3’).

La PCR nichée amplifie une région interne de 145 pb avec le couple d'amorces TCDR1 (5’-TGTGCTCATGTGTTTAAACT-3’) et TCDR2 (5’-CAAAACTATAGTTAC-CAATTTTG-3’) [7].

Les produits d'amplification de la seconde PCR ont été ensuite digérés par l'enzyme de restriction *ApoI* pour la détection de la mutation ponctuelle du codon 76. Les produits de PCR portant le codon sauvage K76 sont coupés par *ApoI* et se présentent sous forme de deux bandes de 47 et 98 pb tandis que les génotypes mutants présentent une seule bande visible de 145 pb. Pour le témoin sauvage, le clone D6 de *P. falciparum* est utilisé tandis que le clone FCM 29 fait office de témoin mutant.

### Génotypage du codon 86 de *Pfmdr1*

Le fragment du gène *Pfmdr1* contenant le codon 86 a été également amplifié par la technique de la PCR nichée. Le premier couple d'amorces externes MDRA1 (5’-TGT-TGAAAGATGGGTAAAGAGCAGAAA-GAG-3’) et MDRA3 (5’-TAGTTTCT-TATTACATATGACACCACAAACA-3’) amplifie une région de 657 pb.

Le second couple d'amorces internes MDRA2 (5’-GTCAAACGTGCATTTTT-TATTAATGACCATTTA-3’) et MDRA4 (5’-AAAGATGGTAACCTCAGTAT-CAAAGAAGAG-3’) amplifie une région de 560 pb [34].

Les produits d'amplification de la seconde PCR ont ensuite été digérés par l'enzyme de restriction AflII pour la détection du codon mutant N86Y. Pour les isolats de type sauvage N86, le profil de restriction se présente sous forme d'une seule bande visible de 560 pb tandis que le génotype mutant apparaît sous forme de deux bandes visibles de 227 et 333 pb. Pour le témoin sauvage, le clone D6 de *P. falciparum* est utilisé tandis que le clone FCM 29 fait office de témoin mutant.

## Résultats

### Caractéristique de la population étudiée

L’âge moyen (± écart-type) des enfants vus en dépistage actif a été de 7,9 ± 3,7 ans dont 6,9 ± 3,7 ans en 2016 et de 8,6 ± 3,5 ans en 2017. Le sexe ratio était de 0,8 (M/F) pour les 2 périodes de l’étude (Tableau I). La température axillaire moyenne en 2016 a été de 36,8 °C et de 37,1 °C en 2017. Le taux de positivité par le TDR était de 59 % (35/59) en 2016 et de 45 % (37/83) en 2017.

**Tableau I:** Caractéristiques de la population étudiée Characteristics of study population

Variables	2016	2017
	(n = 59)	(n = 83)
**Classe d’âge (ans)**		
< 5	17(29%)	14(17%)
5 - 9	25 (42%)	33 (40%)
10 - 15	17(29%)	36 (43%)
**Moyenne d’âge ± écart-type (ans)**	6,9 ± 3,7	8,6 ± 3,5
**Température axillaire moyenne ± écart-type (°C)**	36,8 ± 0,6	37,1 ± 0,8
**Genre**		
féminin	33 (56%)	46 (55%)
masculin	26 (44%)	37(45%)
**TDR positif**	35 (59%)	37(45%)

### Typage du codon 76 du gène *Pfcrt*

**Figure 2:**
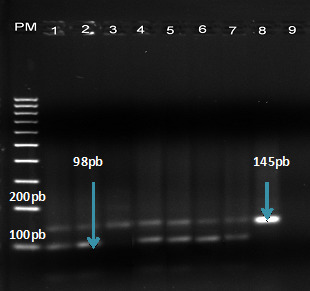
Electrophorèse des produits de digestion de *Pfcrt* avec l'enzyme *ApoI* *Electrophoresis of restriction fragments of* Pfcrt *after digestion with* ApoI

Sur les 142 isolats de *P. falciparum* analysés, 39/142 isolats (27%) ont été amplifiés avec succès pour le gène *Pfcrt* dont 21/59 (35%) en 2016 et 18/83 (22%) en 2017 (Fig. 2). Après la digestion avec l'enzyme de restriction *ApoI,* 38/39 (97,4 %; [IC95% : 84,9 - 99,9]) étaient de génotype sauvage et 1/39 (2,6 %; [IC95% : 0,1 - 15,1]) étaient de génotype mutant (Tableau II).

Source : Auteur

PM : Marqueur de poids moléculaire (100 paires de base) 1, 2, 4,5, 6 : Fragments de *Pfcrt* coupés par *ApoI* chez des isolats sauvages (145 pb : reste du produit d'amplification; 98 pb : produit de restriction après digestion par *ApoI)*

3 : Fragment de *Pfcrt* non coupé par *ApoI* chez un isolat mutant (145 pb)

7 : Clone D6, souche sauvage (K76) coupée par Apo I (K76) (145 pb : reste du produit d'amplification; 98 pb : produit de restriction après digestion par *ApoI)*

8 : Clone FCM29, souche mutante (76T), non coupée par Apo I (76T) de taille égale à 145 pb

**Tableau II:** Fréquence des génotypes pour le codon 76 du gène *Pfcrt* Genotype frequency for the codon 76 of Pfcrt gene

*Pfcrt*	Mutant 76T n (% [IC95%])	Sauvage K76 n (% [IC95%])
**2016 (N = 21)**	1 (4,8 % [IC95% : 0,2 - 25,9])	20(95,2 % [IC95% : 74,1 - 99,7])
**2017 (N = 18)**	0 (0 % [IC95% : 0,0 - 21,9])	18(100 % [IC95% : 78,1 - 100])
**Total (N = 39)**	1 (2,6 % [IC95% : 0,1 - 15,1])	38(97,4 % [IC95% : 84,9 - 99,9])

IC95% : Intervalle de confiance à 95 %

### Typage du codon 86 du gène *Pfmdr1*

**Figure 3:**
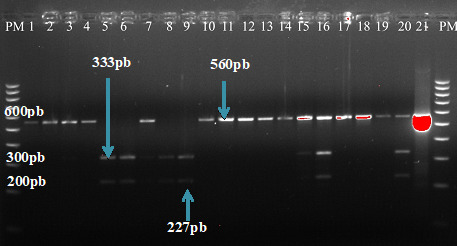
Electrophorèse des produits de digestion de *Pfmdr1* avec l'enzyme AflIII *Electrophoresis of restriction fragments of* Pfmdr1 *after digestion with AfIII*

Parmi les 142 isolats de *P. falciparum* analysés, 56 (39%) ont été amplifiés avec succès pour le gène *Pfmdr1,* dont 28/59 (47%) en 2016 et 28/83 (34%) en 2017

(Fig. 3). Le profil de restriction après la digestion avec AflIII a montré que 36 isolats sur les 56 analysés, soit 64 % [IC95% : 50,3 - 76,3], étaient du type sauvage et 20/56, soit 36 % [IC95% : 23,7 - 49,7], étaient du type mutant (Tableau III).

Source : Auteur PM : Marqueur de poids moléculaire (100 paires de base)

5,6,8,9,15,16 : Fragments de *Pfmdr1* coupés par AflIII, de taille respective de 227 et 333 pb, chez des isolats mutants;

1,2,3,4,7,10,11,12,13,14,17,18,19 : Fragments de *Pfmdr1* non coupés par AflIII, de taille de 560pb, chez des isolats sauvages

20 : Clone FCM29, souche mutante (86Y), coupée par AflIII de 227 et 333 pb et reste de produits d'amplification à 560 pb

21 : Clone D6, souche sauvage (N86), non coupée par AflIII de 560 pb

**Tableau III:** Fréquence des génotypes pour le codon 86 du gène *Pfmdr1* Genotype frequency for the codon 86 of Pfmdr1 gene

*Pfmdr1*	Mutant 86Y n (% [IC95%])	Sauvage N86 n (% [IC95%])
**2016 (N = 28)**	10 (36 % [IC95% : 19,3 - 55,9])	18 (64 % [IC95% : 44,1 - 80,7])
**2017 (N = 28)**	10 (36 % [IC95% : 19,3 - 55,9])	18 (64 % [IC95% : 44,1 - 80,7])
**Total (N = 56)**	20 (36 % [IC95% : 23,7 - 49,7])	36 (64 % [IC95% : 50,3 - 76,3])

IC95% : Intervalle de confiance à 95 %

## Discussion

La technique PCR-RFLP a été utilisée pour la détection des mutations ponctuelles au niveau du codon 76 du gène *Pfcrt* et du codon 86 de *Pfmdr1.* Pour le génotypage des marqueurs moléculaires de la résistance aux antipaludiques, les avantages de la PCR-RFLP comportent son faible coût et sa simplicité lors de l'analyse et l'interprétation des résultats [2]. Ainsi, la PCR-RFLP est utilisée en particulier dans les laboratoires des pays en développement et permet la prise de décision rapide pour ré-orienter et/ou améliorer les stratégies nationales de lutte contre le paludisme. Cependant, d'après nos résultats le taux d'amplification des gènes *Pfcrt* et *Pfmdr1* est de l'ordre de 28 % pour *Pfcrt,* et de 39 % pour *Pfmdr1.* Par rapport aux autres études effectuées chez des enfants selon la même technique, le taux de succès d'amplification de ces gènes d'intérêts est faible [5, 13]. Plusieurs facteurs peuvent affecter la performance de la PCR comme la nature et la quantité de prélèvement (sang total/sang capillaire collecté sur papier buvard), le procédé d'extraction de l'ADN, la parasitémie, la durée et les conditions de stockage des prélèvements, etc. [9]. Un rendement élevé en ADN est crucial particulièrement dans le cas des infections infra-microscopiques ou sous-patentes (infection de faible densité détectable par des méthodes moléculaires, mais pas par le diagnostic de terrain comme la microscopie ou le TDR) où la parasitémie est généralement faible [32], afin d'optimiser les protocoles d’études de génotypages ou des marqueurs de résistance aux antipaludiques [9]. Il a été démontré que la PCR-RFLP ne peut pas détecter les souches minoritaires et les modifications du nombre de copies de gènes [12]. D'autres méthodes réalisables et plus sensibles que la PCR-RFLP pourraient être utilisées comme le HRM (High Resolution Melting). Ce dernier permet de détecter les SNP (Single Nucleotide Polymorphisms ou mutations) associés aux gènes de résistance des antipaludiques mais nécessite l'investissement en thermocycleurs permettant la détection en temps réel [2, 31]. Les enfants de moins de 15 ans pourraient refléter l'image des souches circulantes de *P. falciparum* dans cette localité du fait qu'ils n'effectuent que rarement des déplacements, contrairement aux adultes.

### Typage du codon 76 de *Pfcrt*

La mutation ponctuelle sur le codon 76 du gène *Pfcrt* permet à *P. falciparum* de limiter l'accumulation de la CQ dans sa vacuole digestive, où elle exerce son action inhibitrice. Ce gène est également impliqué dans la baisse de sensibilité de *P. falciparum* à l'amodiaquine et à la quinine [15]. Nos résultats ont montré la présence d'un isolat, soit environ 3 % des isolats analysés, de type mutant au gène *Pfcrt.* Par rapport à d'autres études effectuées à Madagascar, nos résultats sont similaires à ceux observés dans une étude effectuée à Andapa et Tsiroanomandidy entre 2001 et 2002 où la fréquence de l'allèle *Pfcrt* K76T mutant était de 3,3 % [27]. Cependant, une étude effectuée entre 2006 et 2008 au niveau des sites sentinelles de fièvre (926 isolats de Madagascar) a montré une faible prévalence de la mutation au niveau du gène *Pfcrt* de 0,4 % [2]. Récemment en 2018, parmi les 82 isolats analysés d'Ankazomborona (district de Marovoay Nord-Ouest) et de Matanga (district de Vangaindrano Sud-Est), aucune mutation au niveau du gène *Pfcrt* n'a été observée [6]. Contrairement à d'autres pays endémiques d'Asie du Sud-Est ou d'Afrique de l'Est, la prévalence de la mutation du gène *Pfcrt* à Madagascar est faible [27], alors que le taux d’échec thérapeutique de la CQ à Madagascar atteignait 44 % en 2006, ce qui a induit le retrait de cette molécule pour le traitement du paludisme non compliqué à Madagascar [2, 16]. D'ailleurs, il a été démontré que les échecs de traitement à la CQ à Madagascar ont été significativement associés à la mutation au niveau de l'allèle de *Pfmdr1* 86Y [3]. Des études ont montré que le retrait de la chloroquine avait permis le regain des souches de *P. falciparum* sensibles à la CQ [14, 31]. Aux archipels des Comores, par exemple, une augmentation significative de la fréquence des souches sauvages en *Pfcrt,* jusqu’à 76 %, a été observée entre 2006 et 2014 [11]. Cependant, dans certains pays où le paludisme est endémique, malgré le changement de la politique de traitement, les allèles mutants associés à la résistance à la CQ persistent [20], d'où l'intérêt de la surveillance.

### Typage du codon 86 de *Pfmdr1*

La protéine Pgh (produit d'expression du gène *Pfmdr1)* est impliquée dans la modulation de la sensibilité de *P. falciparum* aux antipaludiques comme la CQ, l'AQ, la luméfantrine, la méfloquine etc. Les mécanismes de résistance sont liés soit à une augmentation de l'expression de la protéine, soit à l'apparition de mutations au niveau de ce gène. La mutation au niveau du codon 86 du gène Pfmdr (N86Y) est associée à la résistance à la chloroquine et à l'amodiaquine quand elle est combinée avec la mutation au niveau du codon 76 de *Pfcrt.* D'ailleurs, le seul isolat mutant pour le codon 76 de *Pfcrt* que nous avons détecté est aussi porteur de la mutation au niveau du codon 86 de *Pfmdr1.* Comme la prévalence de ces marqueurs dans la population pourrait indiquer le niveau de la résistance ou de la tolérance face aux médicaments partenaires comme l'AQ et la luméfantrine [19], la surveillance des marqueurs de la résistance aux antipaludiques doit s’étendre sur les molécules partenaires des dérivés d'artémisinine. Parmi les isolats de *P. falciparum* analysés pendant cette étude, 36 % étaient de génotype mutant pour *Pfmdr1* aussi bien pour l'année 2016 que

2017. Une étude effectuée en 2006 dans 3 sites sentinelles de Madagascar a montré que 67,5 % des isolats étaient de type mutant à *Pfmdr1* 86Y [28]. Entre 2006 et 2008, sur des échantillons collectés dans le cadre de la surveillance de la résistance aux antipaludiques à Madagascar, la prévalence de la mutation en *Pfmdr1* codon 86 a été de 21,8 % en 2006 et de 30 % en 2007 [2]. Entre 2009 et 2012, la prévalence de la mutation sur le codon 86 du gène *Pfmdr1* était de 41,4 % à 83,3 % chez des écoliers [1]. Enfin en 2018, chez des enfants de moins de 15 ans dans le district de Marovoay et Vangaindrano, la prévalence de *Pfmdr1* mutant était de 82 % [6]. Aux Comores, après 7 ans de retrait de la CQ, une diminution du taux de *P. falciparum* mutant pour *Pfmdr1* a été observée allant de 87 % à 40 % en 2014 [11].

surveiller les autres codons, à savoir 184 et 1246 du gène *Pfmdr1,* impliqués dans la résistance de *P. falciparum* à l'amodiaquine en Afrique.

## Remerciements

Nous remercions le Fonds mondial pour son soutien financier ainsi que tout le personnel du laboratoire de parasitologie du PNLP pour leur support technique.

## Liens D'intérêt

Les auteurs ne déclarent aucun conflit d'intérêts.

## Conclusion

Malgré le nombre limité d’échantillons analysés, notre étude a mis en évidence la circulation d'isolats portant à la fois la mutation au niveau du codon 76 de *Pfcrt* et 86 de *Pfmdr1.* Le taux de mutation que nous avons observé est faible. D'autres études méritent d’être effectuées afin de suivre l’évolution de ces marqueurs dans le temps et dans l'espace (dans différentes localités). L'utilisation de méthodes plus sensibles permettrait de mieux caractériser les souches de *P. falciparum* circulant à Madagascar. Étant donné que l'ASAQ est utilisée en première intention pour le traitement du paludisme non compliqué dans le pays, il est également nécessaire de

## Éthique

Dans le cadre de la surveillance de l'efficacité thérapeutique des antipaludiques, cette étude a reçu l'accord du Comité national de recherche biomédicale du Ministère de la santé de Madagascar (No. 083/MSANP/CE/11-2012).

## Contribution Des Auteurs

ER, VHIA, RR, VJ et AR ont contribué à la conception de l’étude et à l'analyse des données. ADR, TAR, FR, RHR et DADR

ont réalisé les analyses moléculaires. Tous les auteurs ont participé à la rédaction et ont approuvé la version finale du manuscrit.
